# Disease activity and damage in patients with primary Sjogren’s syndrome: Prognostic value of salivary gland ultrasonography

**DOI:** 10.1371/journal.pone.0226498

**Published:** 2019-12-31

**Authors:** Vera Milic, Jelena Colic, Andja Cirkovic, Svetlana Stanojlovic, Nemanja Damjanov

**Affiliations:** 1 Institute of Rheumatology, Belgrade, Serbia; 2 Faculty of Medicine, University of Belgrade, Belgrade, Serbia; 3 Department for Medical Statistics and Informatics Faculty of Medicine, Belgrade, Serbia; 4 Clinic for Eye Diseases, Clinical Centre of Serbia, Belgrade, Serbia; Soroka University Medical Center, ISRAEL

## Abstract

**Objectives:**

To assess the association between salivary ultrasonography (sUS) findings and disease activity and damage in patients with primary Sjogren’s syndrome (pSS). We investigated the potential prognostic role of sUS as a tool in the assessment of disease activity.

**Methods:**

In 303 pSS patients, disease activity was assessed by the European League Against Rheumatism (EULAR) Sjogren’s Syndrome Disease Activity Index (ESSDAI), the EULAR Sjogren’s Syndrome Patient Reported Index (ESSPRI), the Sjogren’s Syndrome Disease Activity Index (SSDAI) and the Sjogren’s Syndrome Disease Damage Index (SSDDI). The sUS parenchymal inhomogeneity (de Vita scoring system) was assessed in 303 pSS patients and 111 heathy controls. A receiver operating characteristic (ROC) curve was used to determine the cut-off value of the pathological sUS score. Logistic regression analysis was performed to assess risk factors for moderate and high disease activity.

**Results:**

A pathological sUS score ≥ 2 was recorded in 271 (89.7%) patients and 8 (8.6%) healthy controls. Patients with moderate and high ESSDAI and SSDAI scores had significantly higher US activity in comparison to that of pSS patients with low disease activity (p = 0.006; p = 0.01, respectively). Additionally, pSS patients with moderate and high SSDDI scores had higher US activity (p = 0.031). Pathological sUS correlated with the glandular domain within the ESSDAI and SSDDI (p<0.001). The patients with a severe US score (5–6) had a 3.5 times greater chance of having moderate or high disease activity. The specificity of the severe de Vita sUS score for ESSDAI and SSDAI was 85.1% and 85.2%, respectively. In contrast, the sensitivity of a severe de Vita sUS score for ESSDAI was low, at 29.2%, while the sensitivity for the SSDAI was higher, 42.3%. In the analysis of disease activity, a de Vita score ≥ 5 could be used as a risk factor for moderate and high ESSDAI (p = 0.042) and SSDAI (p = 0.006).

**Conclusions:**

Pathological salivary gland ultrasonography is associated with high disease activity and damage in pSS. Consequently, sUS abnormalities might be surrogate items for glandular domains in the assessment of disease activity and damage. Thus, ultrasonography of the salivary gland combined with clinical and serological markers might be part of the next prognostic and therapeutic algorithm in the near future.

## Introduction

Primary Sjogren’s syndrome (pSS) is a chronic systemic autoimmune disease characterized mainly by symptoms of ocular and oral dryness. However, up to 20% of patients have disease-related extra-glandular manifestations [1]. Autoantibodies to the autoantigens Ro/SS-A and La/SS-B are the most specific biomarkers for pSS, whereas cryoglobulins and hypocomplementaemia are the major prognostic markers of disease activity [2]. These patients are also at increased risk of having associated malignancies, particularly non-Hodgkin’s lymphoma [relative risk (RR)], (RR = 13.76) [3,4].

Treatment of patients with pSS is usually symptomatic (artificial tears and saliva replacement). None of the conventional immunosuppressant therapies are of proven effectiveness for systemic features of the disease. Thus, there is a growing interest in using current biological therapies in the treatment of SS [5–7]. In order to define key inclusion and response criteria in clinical trials with biologics, it is important to have objective measures of both disease activity and disease damage. Recently, standardized outcome tools for measuring disease-specific activity and patients’ reported symptoms have been developed by the European League Against Rheumatism (EULAR) SS study group: the EULAR SS Disease Activity Index (ESSDAI) for systemic features of pSS and the EULAR SS Patient-Reported Index (ESSPRI) for patient symptoms [8,9]. The distinction between disease activity (reversible) and damage (irreversible) has always been a matter of debate. For this purpose, two clinical indexes were derived from Italian authors in 2007: Sjogren’s Syndrome Disease Damage Index (SSDDI) for assessment of disease damage and Sjogren’s Syndrome Disease Activity Index (SSDAI) for disease activity [10]. The alterations in salivary glands are important parameters included in both disease activity indexes. The glandular domain in the ESSDAI and the new appearance or increased swelling of major salivary glands in the SSDAI contribute a significant number of points to the total score of disease activity. Apart from the size, the morphological changes in the salivary glands in pSS (either related to disease activity or damage) may be the important components of the clinical indexes. Among the modern imaging techniques, salivary ultrasonography (sUS) has an established role in the diagnosis and follow-up of pSS patients [11–16]. Recently, studies have shown that sUS is able to reveal improved salivary gland echostructure in patients with SS receiving rituximab [17,18]. These results indicate the reversibility of some of the salivary gland changes, most likely reflecting disease activity as opposed to disease-induced damage. Therefore, the presence of salivary gland fibrosis or atrophy detected by sUS could contribute to selecting the subset of pSS patients who are likely not to benefit from immunomodulatory treatment.

The purpose of our study was to assess the association between sUS findings and disease activity and damage in pSS patients. Here, we demonstrated the potential prognostic role of sUS as a tool in the assessment of disease activity.

## Materials and methods

### Participants

The cross-sectional study enrolled 303 pSS patients who fulfilled the American-European Consensus Group (AECG) classification criteria [19]. For sUS evaluation, the control group included 111 healthy subjects without any symptoms of dryness and concomitant autoimmune or thyroid disease. The study was approved by the local Ethics Committee Institute of Rheumatology (number 20/1-51). All patients gave informed written consent.

The questionnaire-based evaluation included the following: ocular symptoms; oral symptoms; ocular signs (Rose Bengal test); salivary gland involvement (sialo-scintigraphy and/or biopsy of minor salivary glands (MSGs) evaluated by the Chisholm and Mason scale); serological tests, including rheumatoid factor (RF), antinuclear antibody (ANA), and antibodies to the extractable nuclear antigens SS-A and SS-B; symptoms/signs suggestive of disease-related extra-glandular manifestations; and related current treatments. The sUS examination was performed simultaneously with the other diagnostic procedures.

### Assessment of SS disease activity and damage by clinical indexes

At enrolment, physicians completed the ESSDAI for each pSS patient. The ESSDAI (0–123) proposes the evaluation of 12 domains or organ systems (constitutional, lymphadenopathy, glandular, articular, cutaneous, pulmonary, renal, peripheral nervous system, central nervous system, muscular, haematological and biology). Low activity (ESSDAI<5), moderate activity (5≤ESSDAI≤13) and high activity (ESSDAI≥14) levels were defined [8]. Physicians completed the Sjogren’s Syndrome Disease Activity Index (SSDAI) and the Sjogren’s Syndrome Disease Damage Index (SSDDI) [10]. The SSDAI is a global score (0–21) including the following items (constitutional, change in salivary gland swelling, articular symptoms, haematologic features, pleuropulmonary symptoms, change in vasculitis, renal involvement and peripheral neuropathy). An SSDAI score ≥5 was defined as a high level of activity [10]. The SSDDI comprises a three-domain assessment (ocular, oral and systemic damage). The systemic domain was further classified into neurological, renal, pulmonary, cardiovascular, gastrointestinal, musculoskeletal, endocrine and malignancy sub-domains. The maximum score for each item was 1, and it has 27 items in total [10].

All patients completed the ESSPRI, a VAS scale (0–10) for dryness, fatigue and pain [9].

### Salivary gland ultrasonography

**Fig 1:**
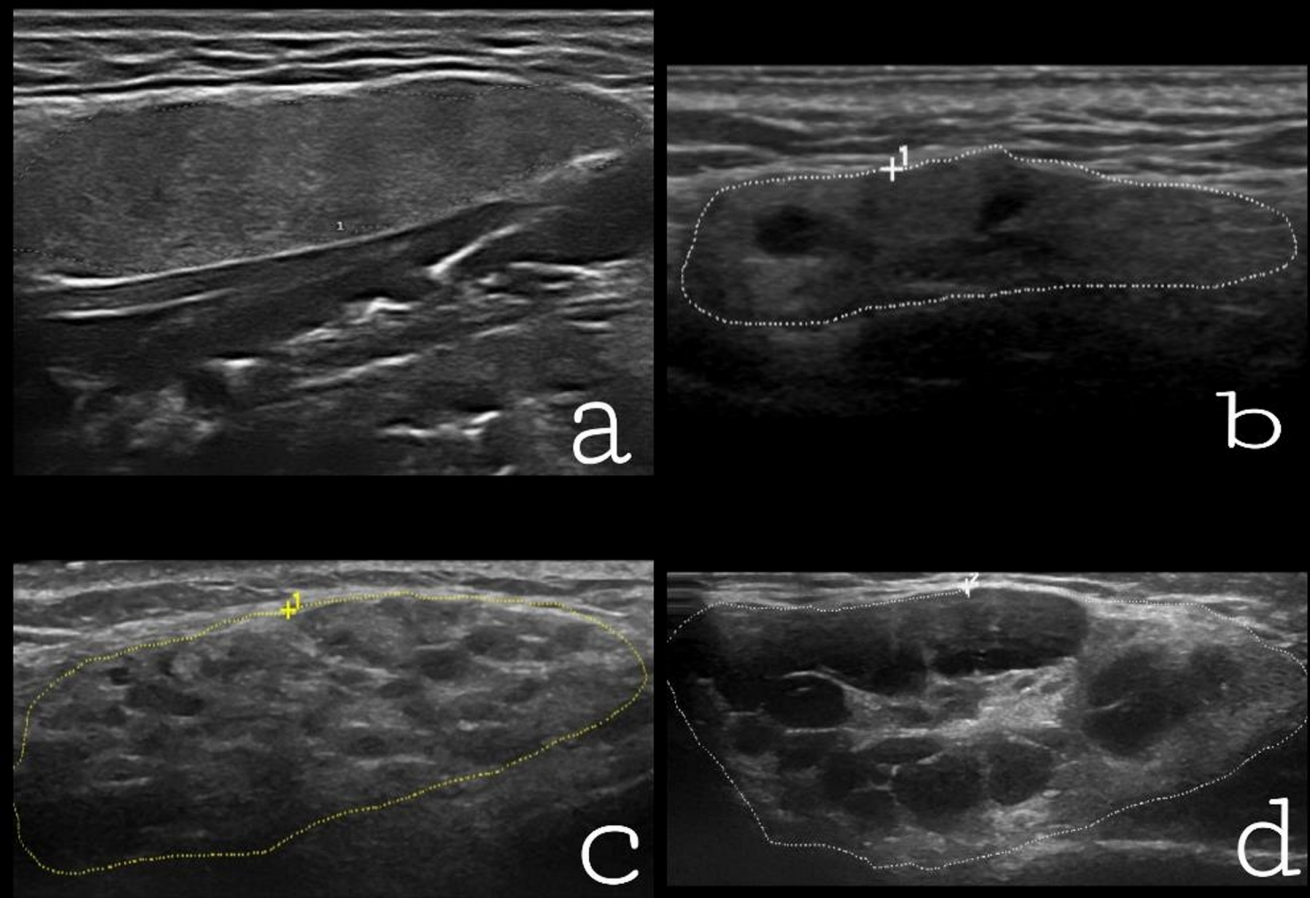
Parenchymal inhomogeneity of the salivary glands demonstrated by ultrasonography. a) Normal salivary gland (grade 0); b) a mild level of salivary inhomogeneity with isolated hypoechoic areas (grade 1); c) evident level of salivary inhomogeneity (grade 2); and d) gross level of salivary inhomogeneity (grade 3).

The parotid and submandibular glands were examined by US using a GE LogiqE9 with a linear high-frequency transducer (6–15 MHz). The parotid glands were evaluated in a longitudinal and cross-sectional plane and the submandibular glands in a longitudinal plane. All ultrasound scans were performed by the same examiner (VM), blinded to the clinical diagnosis. The de Vita scoring system [20] was used for graded changes in the parenchymal homogeneity of salivary glands: grade 0 (normal homogenous parenchyma); grade 1 (mild level of inhomogeneity, with isolated and small hypoechoic areas, without hyperechoic bands); grade 2 (moderate inhomogeneity with multiple hypoechoic areas and/or few hyperechoic bands); grade 3 (severe inhomogeneity with large and confluent hyperechoic areas and diffuse hyperechoic bands) (Fig 1). The sUS score (0–6) represents the sum of the single scores of each pair of parotid and submandibular glands. Disease activity by US was graded into three groups: normal (US score 0–1), moderate (US score 2–4) and severe (US score 5–6).

## Statistical analysis

All statistical analyses were performed using Statistical Package for Social Science (SPSS) software version 21.0. For statistical comparison, Student’s t test, chi-square test, and Fisher’s exact test were used as appropriate. Spearmen's test was used for correlation analysis. A receiver operating characteristic (ROC) curve was used to determine the cut-off value of the pathological sUS score with the highest level of accuracy. We calculated the diagnostic accuracy of a de Vita score ≥ 5 in comparison with ESSDAI, ESSPRI, SSDDI and SSDAI. All possible variables were first analysed through univariate logistic regression, and only significant variables were then summarized in a multivariate logistic regression model. The de Vita score was analysed as a potential predictor with a normal score as the reference. For all statistical analyses, p values less than 0.05 were considered statistically significant.

## Results

### Characteristics of patients with pSS

A total of 303 pSS patients (96.7% females) were enrolled in the study. The control group consisted of 111 healthy controls (97.3% females). There was no statistically significant difference in the mean age between pSS patients and healthy controls (54 ±12 vs.52 ±15, p = 0.131). The general characteristics of pSS patients are presented in Table 1. The frequencies of domains in different ESSDAI and ESSPRI scores are presented in S1 and S2 Tables.

**Table 1:** General characteristics of pSS patients.

Characteristic	pSS patients (N = 303)
Age (years), mean ±SD	54.03±11.95
Female sex, number (%)	293 (96.7)
Disease duration (years), med (min-max)	5 (1–22)
< 5 years, n (%)	128 (43)
5–10 years, n (%)	113 (38)
>10 years, n (%)	59 (20)
Clinical signs	
Ocular symptoms, n (%)	275 (91.4)
Oral symptoms, n (%)	278 (92.4)
Lymphoma, n (%)	8 (2.6)
Diagnostic tests	
Positive keratoconjunctivitis sicca, n (%)	197(96.1)
Positive scialo-scintigraphyɸ, n (%)	150/152 (98.7)
Positive biopsy of MSGɸ, n (%)	190/219 (86.8)
Positive RF, n (%)	212 (70)
Positive ANA, n (%)	223 (73.6)
Positive Anti-SSA Ab, n (%)	254 (83.8)
Positive Anti-SSB Ab, n (%)	153 (50.5)
Disease activity indexes	
ESSDAI, med (min-max), IQR	6 (0–75) 8
ESSDAI ≥5, n (%)	201 (66.3)
ESPPRI, med (min-max), IQR	6 (0–10) 2.67
ESPPRI ≥5, n (%)	219 (72.3)
SSDAI, med (min-max), IQR	5 (0–18), 3
SSDAI ≥5, n (%)	160 (53.2), 3
SSDDI med (min-max), IQR	2 (0–12)
Current treatments	
Glucocorticoids, n (%)	161 (53.1)
Hydroxychloroquine (n%)	225 (74.3)
Azatioprine, n (%)	17 (4.1)
Methotrexate, n (%)	18 (4.3)
Cyclophosphamide, n(%)	3 (1)

Except where indicated otherwise, values are the number n, (%); ESSDAI, EULAR Sjogren’s Syndrome Disease Activity Index; ESSPRI, EULAR Sjogren’s Syndrome Patient Reported Index; EULAR European League Against Rheumatism; SSDAI, Sjogren’s Syndrome Disease Activity Index (SSDAI); SSDDI, Sjogren’s Syndrome Disease Damage Index; MSG: minor salivary glands; RF: rheumatoid factor; ANA: antinuclear antibody; Anti-SSA Ab, anti-SSA antibody; Anti-SSB Ab, anti-SSB antibody.

ɸ Values of objective tests given as rates of positive results (positive/total)

### Ultrasonography findings and correlations

In comparison to healthy controls, the calculated sUS AUC-ROC was 0.96 (0.009) (95% CI 0.94–0.97), which reached a range of very high accuracy. The optimal cut-off for sUS score was set at ≥ 2, with the best ratio of sensitivity (89.6%) and specificity (86.7%). Two hundred seventy-one (89.7%) patients with pSS and only 8 (8.6%) healthy controls had sUS scores ≥ 2. Out of 271 pSS patients with pathological sUS, 197 (65.2%) had moderate sUS activity, and 74 (24.5%) had severe sUS activity.

The overall sUS score correlated directly with the age of patients (r = 0.09, p = 0.05), biopsy of MSG (r = 0.17, p = 0.01), SSDDI (r = 0.16, p = 0.003), SSDAI (r = 0.22, p<0.0001) and ESSDAI (r = 0.428, p<0.0001). Furthermore, pathological sUS correlated with the constitutional domain (r = 0,16, p = 0.004), lymphadenopathy (r = 0.25, p<0.001), glandular domain (r = 0,25, p<0.001) and muscular domain (r = 0,16, p = 0.008) within the ESSDAI. Additionally, pathological sUS correlated with the glandular domain within the SSDDI (r = 0,16, p = 0.004). Disease duration and ESSPRI did not correlate with sUS score (p>0.005).

### Characteristics of pSS patients and ESSDAI and ESPRI disease activity

Table 2 presents the characteristics of pSS patients with different grade ESSDAI and ESSPRI indexes. Patients with moderate and high ESSDAI scores exhibited significantly higher US activity (p = 0.006), were more frequently positive for anti-SSA (p = 0.014) and had more common lymphoma (p = 0.043) in comparison to those of SS patients with low ESSDAI scores. These patients were taking corticosteroids more frequently (p = 0.008), azathioprine (p = 0.003) and methotrexate (p = 0.038). Patients with moderate ESSPRI scores were older (p = 0.024) and more often had both xerophthalmia (p = 0.003) and xerostomia (p = 0.022) in comparison to those of pSS patients with low disease activity.

**Table 2:** Characteristics of pSS patients and comparison according to the level of disease activity using the ESSDAI and ESSPRI.

Characteristic	ESSDAI		p value	ESSPRI		p value
	Low (n = 101)	Moderate and high (n = 202)		Low (n = 83)	Moderate (n = 220)	
Age, mean±SD	55.19±12.02	53.68±11.89	0.299	51.48±13.10	55.20±11.33	0.024
Female, n (%)	100 (98.0)	194 (96.0)	0.356	80 (96.4)	214 (96.8)	0.846
Duration of disease, med (min-max)	5 (1–20)	5 (0–22)	0.456	5 (1–22)	5 (0–20)	0.828
Xerophthalmia, n (%)	94 (92.2)	183 (90.6)	0.651	69 (83.1)	208 (94.1)	0.003
Xerostomia, n (%)	94 (91.2)	186 (92.1)	0.906	72 (86.7)	208 (94.5)	0.022
Positive keratoconjunctivitis sicca, n (%)	65 (95.6)	133 (96.4)	0.783	59 (96.7)	139 (95.9)	0.771
Positive sialo-scintigraphy, n (%)	47 (97.9)	103 (99.0)	0.533f	39 (100.0)	111 (98.2)	0.651
Positive biopsy of MSG, n (%)	55 (84.6)	136 (88.3)	0.381	45 (86.5)	146 (87.4)	0.787
De Vita score (US activity), n (%)			0.006			0.233
Normal (0–1)	16 (15.8)	16 (7.9)		12 (14.5)	20 (9.1)	
Moderate (2–4)	70 (69.3)	127 (62.9)		55 (66.3)	142 (64.5)	
Severe (5–6)	15 (14.9)	59 (29.2)		16 (19.3)	58 (26.4)	
Positive RF, n (%)	78 (77.2)	157 (78.1)	0.862	66 (79.5)	169 (77.2)	0.661
Positive ANA, n(%)	73 (72.3)	149 (73.8)	0.783	64 (77.1)	158 (71.8)	0.353
Positive anti-SSA, n (%)	82 (88.2)	187 (95.9)	0.014	73 (94.8)	196 (92.9)	0.562
Positive anti-SSB, n (%)	45 (44.6)	107 (53.0)	0.167	44 (53.0)	108 (49.1)	0.543
Corticosteroids, n (%)	43 (42.6)	118 (58.7)	0.008	39 (47.6)	122 (55.5)	0.221
Hydroxychloroquine, n (%)	77 (74.3)	149 (74.1)	0.981	64 (78.0)	160 (72.7)	0.347
Azatioprine, n (%)	0 (0)	16 (8.0)	0.003	5 (6.1)	11 (5.0)	0.771
Methotrexate, n (%)	2 (2.0)	16 (8.0)	0.038	2 (2.4)	16 (7.3)	0.115
Cyclophosphamide, n (%)	0 (0)	3 (1.5)	0.217	0 (0)	3 (1.4)	0.565f
Lymphoma, n (%)	0 (0)	8 (4.0)	0.043	3 (3.6)	5 (2.3)	0.516

Low activity: ESSDAI<5; Moderate to high activity: 5≥ESSDAI or Low activity: ESSPRI<5; Moderate activity≥5. Except where indicated otherwise, values are the number (%); ESSDAI, EULAR Sjogren’s Syndrome Disease Activity Index; ESSPRI, EULAR Sjogren’s Syndrome Patient Reported Index; EULAR European League Against Rheumatism; MSG minor salivary gland; RF rheumatoid factor; ANA antinuclear antibody; Anti-SSA Ab, anti-SSA antibody; Anti-SSB Ab, anti-SSB antibody;

### Characteristics of pSS patients and SSDDI and SSDAI disease activity

Table 3. presents the characteristics of pSS patients with different grades of the SSDDI and SSDAI. Patients with moderate and high SSDDI scores showed higher US activity (p = 0.031) and more common lymphoma (p<0.001) compared to SS patients with low disease activity. Patients with moderate SSDAI scores more frequently had positive biopsies of MSG (p = 0.015), positive anti-SSB (p = 0.033), concurrent therapy of corticosteroids (p = 0.019) and higher US activity (p = 0.01) compared to those of pSS patients with low disease activity.

**Table 3:** Characteristics of pSS patients and comparison according to the level of its activity using SSDDI and SSDAI scores.

Characteristic	SSDDI		p value	SSDAI		p value
	Low (n = 277)	Moderate and high (n = 26)		Low (n = 142)	Moderate (n = 161)	
Age, mean±SD	54.13±12.17	54.73±9.24	0.808	54.71±11.99	53.71±11.90	0.467
Female, n (%)	270 (97.1)	24 (92.3)	0.207f	140 (97.9)	154 (95.7)	0.272
Duration of disease, med (min-max)	5 (0–22)	7 (1–17)	0.059	5 (1–20)	5 (0–22)	0.404
Xerophthalmia, n (%)	253 (91.0)	24 (92.3)	0.824	129 (90.2)	148 (91.9)	0.600
Xerostomia, n (%)	255 (91.9)	25 (96.2)	0.451	131 (92.2)	149 (92.5)	0.923
Positive keratoconjunctivitis sicca, n (%)	182 (96.3)	16 (94.1)	0.504f	90 (96.8)	108 (95.6)	0.658
Positive sialo-scintigraphy, n (%)	139 (98.6)	11 (100.0)	1.000f	68 (98.6)	82 (98.8)	1.000f
Positive biopsy of MSG, n (%)	173 (88.7)	18 (78.3)	0.150	80 (81.6)	111 (92.5)	0.015
De Vita score (US activity), n (%)			0.031			0.001
Normal (0–1)	32 (11.6)	0 (0.0)		18 (12.7)	14 (8.7)	
Moderate (2–4)	182 (65.7)	15 (57.7)		103 (72.5)	94 (58.4)	
Severe (5–6)	63 (22.7)	11 (42.3)		21 (14.8)	53 (32.9)	
Positive RF, n (%)	215 (77.9)	20 (76.9)	0.909	106 (75.2)	129 (80.1)	0.302
Positive ANA, n(%)	231 (83.4)	24 (92.3)	0.234	116 (81.7)	139 (86.3)	0.269
Positive anti-SSA, n (%)	244 (93.1)	25 (96.2)	0.554	121 (91.0)	148 (95.5)	0.125
Positive anti-SSB, n (%)	140 (50.5)	12 (46.2)	0.669	62 (43.7)	90 (55.9)	0.033
Corticosteroids, n (%)	145 (52.5)	16 (61.5)	0.379	65 (46.1)	96 (59.6)	0.019
Hydroxychloroquine, n (%)	205 (74.3)	19 (73.1)	0.894	102 (72.3)	122 (75.8)	0.496
Azatioprine, n (%)	13 (4.7)	3 (11.5)	0.223f	4 (2.8)	12 (7.5)	0.120f
Methotrexate, n (%)	17 (6.2)	1 (3.8)	0.634	11 (7.8)	7 (4.3)	0.206
Cyclophosphamide, n (%)	2 (0.7)	1 (3.8)	0.237f	2 (1.4)	1 (0.6)	0.486
Lymphoma, n (%)	1 (0.4)	7 (26.9)	<0.001	2 (1.4)	6 (3.7)	0.209

Except where indicated otherwise, values are the number (%); SSDAI, Sjogren’s Syndrome Disease Activity Index; SSDDI, Sjogren’s Syndrome Disease Damage Index; MSG minor salivary gland; RF rheumatoid factor; ANA antinuclear antibody; Anti-SSA Ab, anti-SSA antibody; Anti-SSB Ab, anti-SSB antibody; according to the chi-square test or Fisher`s exact test where appropriate (f)

### Predictive value of clinical, serological and sUS variables for moderate and high pSS activity and damage

The results of logistic regression analyses (Table 4) showed that severe US activity is an independent predictor of moderate and high pSS activity according to the ESSDAI score. The patients with a severe de Vita score (5–6) had a 3.5 times greater chance of having moderate or high disease activity. The presence of lymphoma increased extremely, with a 263.41-fold higher risk for moderate disease activity according to SSDAI. SSDAI greater than or equal to 5 highly correlated with positive biopsy of MSG (OR 3.061).

**Table 4:** Odds ratios from multivariate logistic regression models for possible clinical, serological and sUS variables as predictors for moderate and high pSS activity assessed by clinical indexes.

Predictor	ESSDAI Moderate and high		ESSPRI Moderate and high		SSDDI Moderate		SSDAI Moderate	
	OR	P	OR	P	OR	P	OR	P
De Vita score Normal (0–1)	ref	ref			ref	ref	ref	ref
De Vita score Moderate (2–4)	1.598	0.232			NA	0.998	0.588	0.350
De Vita score Severe (5–6)	3.556	0.007			NA	0.998	1.230	0.747
Age			1.013	0.299				
Xerophthalmia			2.075	0.113				
Xerostomia			2.147	0.116				
Lymphoma					263.428	<0.001		
Positive of biopsy of MSG							3.061	0.020
Positive anti-SSB							1.585	0.133
Glucocorticoids							1.533	0.153

ESSDAI, EULAR Sjogren’s Syndrome Disease Activity Index; ESSPRI, EULAR Sjogren’s Syndrome Patient Reported Index; EULAR European League Against Rheumatism; SSDAI, Sjogren’s Syndrome Disease Activity Index (SSDAI); SSDDI, Sjogren’s Syndrome Disease Damage Index; MSG minor salivary gland; Anti-SSB Ab, anti-SSB antibody;

The diagnostic accuracy of a de Vita score ≥ 5 in comparison with ESSDAI, ESSPRI, SSDDI and SSDAI ≥ 5 is presented in Table 5. The specificity of a severe de Vita sUS score for ESSDAI and SSDAI was 85.1% and 85.2%, respectively. In contrast, the sensitivity of a severe de Vita sUS score for ESSDAI was low, 29.2%, while the sensitivity for SSDAI was higher, 42.3%. In the analysis of disease activity, a de Vita score ≥ 5 could be a risk factor for moderate and high ESSDAI (p = 0.042) and SSDAI (p = 0.006). However, this could not be applied for ESSPRI and SSDDI (p>0.05).

**Table 5:** Diagnostic accuracy of a de Vita score ≥5 in comparison with ESSDAI, ESSPRI, SSDDI and SSDAI≥5.

De Vita score vs.	ESSDAI	ESSPRI	SSDDI	SSDAI
Sensitivity	29.2% (95% CI 23.0–36.0)	26.4% (95% CI 20.7–32.7)	42.3% (95% CI 23.4–63.1)	32.9% (95% CI 25.7–40.8)
Specificity	85.1% (95% CI 76.7–91.4)	80.7% (95% CI 70.6–88.6)	77.3% (95% CI 71.9–82.1)	85.2% (95% CI 78.3–90.6)
Overall accuracy	47.9% (95% CI 42.1–53.6)	41.3% (95% CI 35.7–47.0)	74.3% (95% CI 68.9–79.1)	57.4% (95% CI 51.6–63.1)
Positive predictive value	79.7% (95% CI 68.8–88.2)	78.4% (95% CI 67.3–87.1)	14.9% (95% CI 7.7–25.0)	71.6% (95% CI 59.9–81.5)
Negative predictive value	37.6% (95% CI 31.3–44.2)	29.3% (95% CI 23.5–35.6)	93.4% (95% CI 89.4–96.3)	52.8% (95% CI 46.2–59.4)
Likelihood ratio +	1.97 (95% CI 1.18–3.29)	1.37 (95% CI 0.84–2.24)	1.86 (95% CI 1.13–3.06)	2.23 (95% CI 1.42–3.50)
Likelihood ratio -	0.83 (95% CI 0.73–0.94)	0.91 (95% CI 0.80–1.04)	0.75 (95% CI 0.53–1.04)	0.79 (95% CI 0.69–0.89)
Area under the ROC	57.2%	53.5	59.8	59.1
P	0.042	0.341	0.099	0.006

ESSDAI, EULAR Sjogren’s Syndrome Disease Activity Index; ESSPRI, EULAR Sjogren’s Syndrome Patient Reported Index; EULAR European League Against Rheumatism; SSDAI, Sjogren’s Syndrome Disease Activity Index (SSDAI); SSDDI, Sjogren’s Syndrome Disease Damage Index.

## Discussion

In the clinical setting, the assessment of disease activity indexes is essential in patients with pSS. The relationship between different sUS scores and disease severity has been recently demonstrated [21–23]. However, these studies have focused only on EULAR indexes or associations between some of the clinical aspects of pSS with sUS damage. In our study, we tested the correlation of sUS using sets of indexes for disease activity developed by both Vitali et al. and the EULAR group. We found that a higher sUS score was associated with higher ESSDAI and SSDAI indexes of disease activity, with an estimated US diagnostic specificity for ESSDAI and SSDAI of 85.1% and 85.2%, respectively. To our knowledge, this was not previously reported.

The predictive value of different factors for disease activity, including parenchymal inhomogeneity as the sUS hallmark for pSS, has been demonstrated [24,25]. Inhomogeneity of the salivary parenchyma detected by sUS includes the presence of hypoechoic areas and/or hyperechoic bands [26]. These sUS changes indicate active glandular inflammation due to the infiltration of immune cells and/or chronic damage with fibrotic lesions and loss of functional parenchyma [27]. Several sUS scoring systems have been proposed for the evaluation of typical sUS changes in pSS; however, no consensus has been reached yet [25]. In our study, grade 2 of the de Vita scoring system was denoted as abnormal sUS findings, and this cut-off value had the best diagnostic sensitivity (89.6%) and specificity (86.7%) for pSS, according to ROC curve analyses. Likewise, Jouse-Joulin et al. [28] reported that the diagnostic sensitivity of sUS for pSS ranged from 45.8% to 91.6% and that the specificity ranged from 73% to 98.1%.

Interestingly, in our cohort, 15 out of 202 (7.5%) pSS patients with ESSDAI ≥5 had normal sUS. The preserved US structure of the salivary glands with functional impairment in these cases indicates that other mechanisms may be involved in the pathogenesis of pSS, including abnormalities in parasympathetic neurotransmission [29]. In our cohort, 43% of the pSS patients had a duration of disease <5 years, while 89.7% of these patients had pathological sUS. We found no correlation between disease duration and sUS change, similar to Theander E et al. [16]. This finding implies that changes in the salivary gland parenchymal echostructure are likely to be present in the early course of disease. On the other hand, we found positive correlations between sUS findings and indexes of disease activity and damage as well as serological tests, lymphoma and immunosuppressive therapy. Consistent with our findings, Fidelix et al. [15] reported an association of more severe sUS scores with an ESSDAI ≥ 5 and serological tests. Additionally, Kimura-Hayama E et al. [30] have also reported that elastography ultrasound of the major salivary glands correlates with the ESSDAI. However, this study did not find a correlation with the ESSPRI, similar to our results.

In our study group, 66.3% of pSS patients exhibited disease activity with an ESSDAI score ≥5, while only 3.1% of pSS patients had no systemic disease activity (ESSDAI = 0). According to Brito-Zeron et al. [31], pSS patients with high systemic disease activity are at high risk of death, and close follow-up (3–6 months) is strongly advised. In our study, analyses of domains of the ESSDAI revealed articular involvement as the most frequent finding (85%), followed by haematological (67.2%) and glandular (44%) involvement. This is in line with previously reported studies [32, 33]. Here, we found that pathological sUS correlated with the glandular domain within the ESSDAI and SSDDI. This is one possible reason for the higher disease activity, so sUS might be a surrogate item for the glandular domain in an objective measure of disease-related damage to salivary glands. Interestingly, disease longevity > 5 years, de Vita scores ≥2, and current therapy with glucocorticoids and methotrexate were identified as variables independently associated with a higher ESSDAI score. In addition, we noted that abnormal sUS was an independent prognostic factor, whereas a severe de Vita score had high predictive value for high and moderate levels of ESSDAI and SSDAI.

However, some limitations of our study are worth noting. A progression of sUS changes in pSS patients could not be observed due to the cross-sectional design of this study. The main limitation of our research is related to the assessment of sUS score by a single ultrasonographer. Although the experienced ultrasonographer was blinded to the diagnosis, it is well known that sUS is a subjective method of imaging.

In conclusion, pathological salivary gland ultrasonography is associated with high disease activity and damage in pSS. Consequently, sUS abnormalities might represent a surrogate item for the glandular domain in the assessment of disease activity and damage. Thus, ultrasonography of the salivary glands combined with clinical and serological markers might be part of the next prognostic and therapeutic algorithm in the near future.

## Supporting information

S1 Table The frequencies of domains in different ESSDAI scores. Low activity: ESSDAI<5; Moderate activity: 5≤ESSDAI≤13: High activity: ESSDAI≥14.
(DOCX)

S2 Table The frequencies of domains in different ESSPRI scores. Absence activity: ESSPRI 0; Low activity ESSPRI 5; Moderate and high activity: ESSPRI high ≥5.
(DOCX)

## Abbreviations

pSS: primary Sjogren’s syndrome
sUS: salivary ultrasonography
EULAR: European League Against Rheumatism
ESSDAI: EULAR Sjogren’s Syndrome Disease Activity Index
ESSPRI: EULAR Sjogren’s Syndrome Patient Reported Index
SSDAI: Sjogren’s Syndrome Disease Activity Index (SSDAI)
SSDDI: Sjogren’s Syndrome Disease Damage Index
MSG: minor salivary gland
RF: rheumatoid factor
ANA: antinuclear antibody
Ab: antibody
Anti-SSA Ab: anti-SSA antibody
Anti-SSB Ab: anti-SSB antibody
