# Supplementary material for: Disease activity and damage in patients with primary Sjogren’s syndrome: Prognostic value of salivary gland ultrasonography
Source: PLoS One. 2019 Dec 31;14(12):e0226498. doi: 10.1371/journal.pone.0226498 (PMC6938326; doi:10.1371/journal.pone.0226498)
Supplement: S1 Table — Low activity: ESSDAI<5; Moderate activity: 5≤ESSDAI≤13: High activity: ESSDAI≥14. (DOCX) [file pone.0226498.s001.docx]

| ESSDAI domains (weight factor) | Absence  Activity | Low  Activity | Moderate and high  activity |
| --- | --- | --- | --- |
| Constituonal domain (3) | 264 (87.4%) | 34 (11.3%) | 4 (1.3%) |
| Lymphadenopathy domain (4) | 258 (85.7%) | 30 (10%) | 13 (4.3%) |
| Glandular domain (2) | 168 (55.8) | 81 (26.9%) | 52 (17.3%) |
| Articular domain (2) | 44 (14.6%) | 180 (59.8%) | 77 (25.6%) |
| Cutaneous domain (3) | 237 (78.7) | 16 (5.3%) | 48 (16%) |
| Pulmonary domain (5) | 264 (88%) | 24 (8%) | 12 (4.0%) |
| Renal domain (5) | 294 (97.7%) | 5 (1.7%) | 2 (0.7%) |
| Muscular domain (6) | 271 (90%) | 26 (8.6%) | 4 (1.4%) |
| Peripheral nervous system domain (5) | 249 (82.7%) | 46 (15.3%) | 6 (2%) |
| Central nervous system domain (5) | 292 (97%) | 6 (2%) | 3 (1%) |
| Haematologycal domain (2) | 234 (77.7%) | 64 (21.3%) | 3 (1%) |
| Biologycal domain (1) | 258 (85.7%) | 30 (10%) | 13 (4.3%) |
