# Supplementary material for: Disease activity and damage in patients with primary Sjogren’s syndrome: Prognostic value of salivary gland ultrasonography
Source: PLoS One. 2019 Dec 31;14(12):e0226498. doi: 10.1371/journal.pone.0226498 (PMC6938326; doi:10.1371/journal.pone.0226498)
Supplement: S2 Table — Absence activity: ESSPRI 0; Low activity ESSPRI 5; Moderate and high activity: ESSPRI high ≥5. (DOCX) [file pone.0226498.s002.docx]

| ESSPRI domains (weight factor) | Absence  Activity | Low  Activity | Moderate and high  activity |
| --- | --- | --- | --- |
| Dryness (1) | 3 (1.0%) | 48 (15.8%) | 253 (83.2%) |
| Fatigue (1) | 3 (1.0%) | 48 (15.8%) | 253 (83.2%) |
| Pain (1) | 2 (0.7%) | 27 (8.9%) | 275 (90.5%) |
